# Efficacy of chloroquine versus lopinavir/ritonavir in mild/general COVID-19 infection: a prospective, open-label, multicenter, randomized controlled clinical study

**DOI:** 10.1186/s13063-020-04478-w

**Published:** 2020-07-08

**Authors:** Xi Liu, Huili Chen, Yuqi Shang, Hongqiong Zhu, Gongqi Chen, Yuanli Chen, Shaoxuan Liu, Yaoyong Zhou, Mingxing Huang, Zhongsi Hong, Jinyu Xia

**Affiliations:** 1 Department of Infectious Diseases, The Fifth Affiliated Hospital, Sun Yat-sen University, Zhuhai, China; 2 Department of Hospital Infection Control, The Fifth Affiliated Hospital, Sun Yat-sen University, Zhuhai, China; 3 Office of Clinical Research Center, The Fifth Affiliated Hospital, Sun Yat-sen University, Zhuhai, China

**Keywords:** COVID-19, Chloroquine, Lopinavir/ritonavir, Randomized controlled clinical study, Efficacy

## Abstract

**Background:**

The outbreak of COVID-19 (caused by SARS-Cov-2) is very serious, and no effective antiviral treatment has yet been confirmed. The adage “old drug, new trick” in this context may suggest the important therapeutic potential of existing drugs. We found that the lopinavir/ritonavir treatment recommended in the fifth edition of the Treatment Plan of China can only help to improve a minority of throat-swab nucleic-acid results (3/15) in hospitals. Our previous use of chloroquine to treat patients with COVID-19 infection showed an improvement in more throat-swab nucleic-acid results (5/10) than the use of lopinavir/ritonavir.

**Methods/design:**

This is a prospective, open-label, randomized controlled, multicenter clinical study. The study consists of three phases: a screening period, a treatment period of no more than 10 days, and a follow-up period for each participant. Participants with COVID-19 infection who are eligible for selection for the study will be randomly allocated to the trial group or the control group. The control group will be given lopinavir/ritonavir treatment for no more than 10 days. The trial group will be given chloroquine phosphate treatment for no more than 10 days. The primary outcome is the clinical recovery time at no more than 28 days after the completion of therapy and follow-up. The secondary outcomes include the rate of treatment success after the completion of therapy and follow-up, the time of treatment success after no more than 28 days, the rate of serious adverse events during the completion of therapy and follow-up, and the time to return to normal temperature (calculated from the onset of illness) during the completion of therapy and follow-up. Comparisons will be performed using two-sided tests with a statistical significance level of 5%.

**Discussion:**

This experiment should reveal the efficacy and safety of using chloroquine versus lopinavir/ritonavir for patients with mild/general COVID-19 infection. If the new treatment including chloroquine shows a higher rate of throat-swab SARS-CoV-2 real-time fluorescent reverse transcription polymerase chain reaction (RT-PCR) negativity and is safe, it could be tested as a future COVID-19 treatment.

**Trial registration:**

Chinese Clinical Trial Registry, ID: ChiCTR2000029741. Registered on 11 February 2020.

## Introduction

### Background

In December 2019, patients with unexplained pneumonia appeared in Wuhan, China, and were subsequently identified as a having a novel type of coronavirus. On 30 January 2020, the World Health Organization named it “COVID-19 (Corona Virus Disease-2019, caused by Severe Acute Respiratory Syndrome Corona Virus (SARS-Cov-2)).” The COVID-19 epidemic is now spreading throughout the world.

Coronavirus is a single-stranded, positive-strand RNA (ribonucleic acid) virus with an envelope [1]. There are currently no clinically specific drugs for HCoVs (Human Corona Viruses). Recently, the National Health Commission of China announced a new coronavirus-infection-related pneumonia diagnosis and treatment program (the fifth edition, URL: http://www.nhc.gov.cn/yzygj/s7653p/202002/3b09b894ac9b4204a79db5b8912d4440/files/7260301a393845fc87fcf6dd52965ecb.pdf), which proposed the trial of lopinavir/ritonavir for its antivirus effect [2–4]. Our previous use of this drug combination found that the effect of lopinavir/ritonavir on COVID-19 was unsatisfactory, as it only helps to improve the minority of throat-swab nucleic-acid results (3/15).

Chloroquine is not only used as an antimalarial drug, but is also used for the treatment of autoimmune diseases due to its immunomodulatory activity [5].

Previous studies have shown that chloroquine exerts antiviral effects through the following mechanisms:

1. It can change the pH of acidic organelles [6] (such as endosomes), so inhibits infections such as Borna disease virus [7], avian leukemia virus [8], and Zika virus [9]
2. It can change the glycosylation pattern of the HIV virus gp120 envelope, which inhibits the replication of HIV (human immunodeficiency virus) virus in CD4 + T cells [10]
3. It can effectively inhibit autophagy in the lungs of avian influenza H5N1 mice and reduce the damage to the alveolar epithelium [11]
4. It inhibits viral replication by blocking the autophagy phenomenon induced by the Zika virus, and can cut off vertical infection of the Zika virus from the maternal-fetal pathway [12]

Multiple studies have found that chloroquine has anti-SARS-CoV activity:

1. Chloroquine can inhibit viral replication by reducing the terminal glycosylation of the angiotensin-converting enzyme 2 (ACE2) receptor on Vero E6 cells and interfering with the binding of SARS-CoV to the ACE2 receptor [13, 14]
2. Chloroquine inhibits the replication of HCoV-229E (and SARS-CoV, both belong to the α-group HCoVs) by inhibiting the activation of p38 mitogen-activated protein kinase (MAPK) in the L132 human embryonic-lung-cell line [15]
3. The spike protein (S protein) of SARS-CoV-2 is similar in structure to that of SARS-CoV, and can bind to the ACE2 receptor on the host-cell surface to infect the host epithelial cells [16]
4. Remdesivir (GS-5734) and chloroquine (Sigma-C6628) can effectively inhibit SARS-CoV-2 infection [17]

This study will compare the efficacy, safety, and impact on patient compliance between the chloroquine phosphate regimen with the lopinavir/ritonavir regimen in mild/general COVID-19 infection.

## Methods/design

### Setting

This randomized controlled trial is to be conducted at the Fifth Affiliated Hospital of Sun Yat-sen University, the Ninth People’s Hospital of Dongguan, Zhongshan Second People’s Hospital, and Jiangmen Central Hospital.

**Fig. 1:**
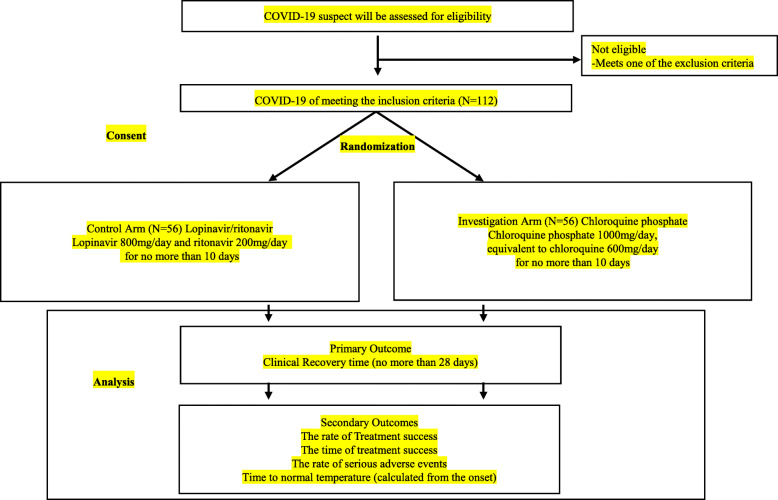
The clinical research flowchart

These hospitals are located in Guangdong, China. The clinical research flowchart (CRF) of the research process is shown in Fig. 1.

Once the volunteered participants have been included, researchers will explain the research procedures in detail and require them to sign a written informed consent form which is signed by the subjects or their legal representative. All participants can withdraw their consent at any time during the trial.

### Design

This is a prospective, open-label, randomized controlled, multicenter clinical study with two arms. Eligible patients are first screened by safety laboratory testing. Patients with diagnosed mild/general COVID-19 infection are randomized to the following two arms at a 1:1 ratio:

1. Arm 1 (control arm): lopinavir/ritonavir treatment for diagnosed mild/general COVID-19 infection uses lopinavir 800 mg/day and ritonavir 200 mg/day for no more than 10 days
2. Arm 2 (investigation arm): chloroquine phosphate treatment using chloroquine phosphate 1000 mg/day, equivalent to chloroquine 600 mg/day for no more than 10 days

The CRF is shown in Fig. 1. The schedule of treatment visits and data collection (also known as the CRF) is shown in Table 1.

**Table 1:**
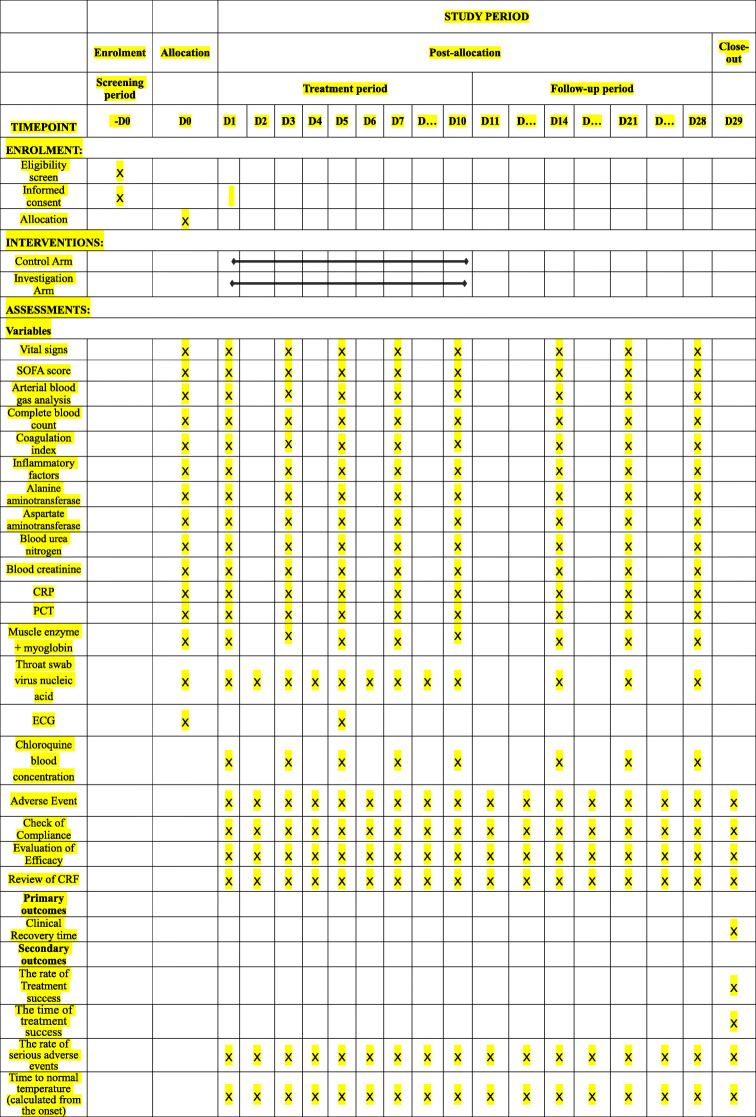
The schedule of treatments and data collection

The *SOFA (Sequential Organ Failure Assessment) score* predicts mortality risk for patients in the intensive care unit based on laboratory results and clinical data^1^

*Coagulation index*: *PT* prothrombin time, *APTT* activated partial prothrombin time, Fib fibrinogen, *D-Dimer* D-dimer, *PLT* platelet count

*Inflammatory factors*: various cytokines involved in inflammation, such as interleukin (IL)-6, IL-10, tumor necrosis factor (TNF)-α, etc.

*PCT (procalcitonin)*: a protein that increases when severe bacterial, fungal, and parasitic infections, sepsis, and multiple organ failure occur

− D0: before the research; D0: start of the research; D1: 1st day of the research; D2: 2nd day of the research; D3: 3rd day of the research; D4: 4th day of the research; D5: 5th day of the research; D6: 6th day of the research; D7: 7th day of the research; D10: 10th day of the research; D14: 14th day of the research; D21: 21st day of the research; D28: 28th day of the research; D29: after the research

References:

1 Medlej K. Calculated decisions: sequential organ failure assessment (SOFA) score. *Emerg Med Pract* 2018;20:CD1-CD2.

Researchers will evaluate treatment adherence during each visit. If a scheduled visit is delayed or cancelled, the research team will contact the participant at once. No treatment or intervention is prohibited for any of the participants.

### Outcomes

The primary outcome is the clinical recovery time of no more than 28 days after the completion of therapy and follow-up. The secondary outcomes include the rate of treatment success after the completion of therapy and follow-up, the time of treatment success after no more than 28 days, the rate of serious adverse events (SAEs) during the completion of therapy and follow-up, and the time to return to normal temperature (calculated from the onset of illness) during the completion of therapy and follow-up.

### Definitions

#### The clinical recovery time and adverse events

We define the clinical recovery time as the time (in hours, no more than 28 days) from the start of study drug intervention to normalization of body temperature, respiratory symptoms (cough, nasal stuffiness, nasal discharge, etc.), respiratory frequency, and blood-oxygen saturation. Specifically, also meeting the following criteria at the same time:

- ① No fever: axillary body temperature ≤ 37.2 °C
- ② Relief of respiratory symptoms (72 consecutive hours)
- ③ Respiration rate ≤ 24/min (resting state)
- ④ Fingertip blood oxygen > 94%

Adverse events (AEs) refer to adverse medical events that occur after a patient or clinical trial participant receives a drug, but they are not necessarily causally related to treatment.

Serious adverse events (SAEs) refer to events that require hospitalization, prolonged hospital stay, disability, affect working ability, are life-threatening or fatal, cause congenital malformation and other events that occur during the clinical trial.

#### Treatment outcomes

We define treatment success as a patient whose throat-swab SARS-CoV-2 real-time fluorescent reverse transcription polymerase chain reaction (RT-PCR) nucleic acid is positive at the beginning of the treatment but is negative at least twice consecutively after the treatment and remains negative.

### Participants

#### Inclusion criteria

1. Age ≥ 18 years
2. Patients diagnosed with COVID-19 according to the criteria of the seventh edition of the Treatment Plan of China for new coronavirus pneumonia (URL: http://202.116.81.74/cache/4/03/www.nhc.gov.cn/5d9aa1423a8a577e1cc197a0d3c434d8/ce3e6945832a438eaae415350a8ce964.pdf):
  ① Epidemiological history:
    - Within 14 days before the onset of illness, a history of travel or residence in Wuhan and surrounding areas, or other communities with reported cases
    - Within 14 days before the onset of illness, exposure to a person with COVID-19 infection (positive nucleic-acid test)
    - Exposure to patients with fever or respiratory symptoms from Wuhan and surrounding areas, or communities with case reports within 14 days before illness onset
    - Aggregated disease: two or more cases of fever or respiratory symptoms occurring in a small area, such as home, office, school class, etc., within 2 weeks
  ② Clinical manifestations:
    - Fever or respiratory-tract symptoms: cough, nasal stuffiness, nasal discharge, etc.
    - Normal or decreased white-blood-cell counts in the early stages of disease; normal or decreased lymphocyte counts
    - Multiple, small, patchy shadows and interstitial changes in the early stages of chest imaging, which are evident in the extrapulmonary zones, and which develop multiple “ground-glass” infiltrations and infiltrates throughout both lungs. In severe cases, pulmonary consolidation may occur, but the formation of pleural effusion is rare
  ③ Confirmed conditions: fulfillment of any one of the epidemiological history items and any two of the clinical manifestation items or three clinical manifestation items if no epidemiological history is available, in addition to meeting one of the following etiological or serological criteria:
    - Real-time fluorescent RT-PCR detects novel coronavirus nucleic acid
    - The gene-sequencing results of patients’ specimens (blood, stool, etc.) are highly homologous to those of known novel coronaviruses
    - Serum SARS-CoV-2-specific antibodies IgM and IgG are positive, serum SARS-CoV-2-specific IgG antibodies change from negative to positive or the quantity of IgG in the recovery phase is at least four times higher than in the acute phase (3–5 days after illness onset)
3. Mild or general patients:
  - Mild: mild clinical symptoms (only manifested as low-grade fever, minimal fatigue, etc.), and no pneumonic manifestations on imaging
  - General: with fever, dry cough and other respiratory-tract symptoms, visible pneumonic imaging)
4. Those who have not used antiviral drugs

#### Exclusion criteria

Any of the following factors will lead to exclusion:

1. Patients with a history of allergy to chloroquine phosphate, lopinavir, or ritonavir
2. Patients with hematological diseases
3. Patients with end-stage liver or kidney diseases
4. Patients with arrhythmia and/or chronic heart disease
5. Patients known to have retinal disease or hearing loss
6. Patients known to have mental illness
7. Patients who must use digitalis because of an original underlying disease
8. Pancreatitis
9. Hemophilia
10. Favism
11. Female patients during pregnancy

### Randomization

Grouping is carried out using a centrally stratified, randomized block method. Before the trial, a statistical expert will use SAS software to set the number of centers at four, the block size will be four, the number of blocks will be 28, using a 1: 1 ratio between the experimental group and the control group, will generate 112 random numbers and corresponding grouping information. According to the haphazard allocation table used in advance, the statistical expert gives random numbers (1–112) in ascending order. Each random number and grouping information correspond to an envelope. The envelope is sealed and given to the researchers responsible for screening. Qualified subjects are selected, and the envelopes are received in the order of enrollment. After the envelopes are opened, the random-number and grouping information is removed, so that the subjects will be randomly assigned to the experimental group or the control group, and the corresponding treatment and observations performed. Each subject’s random number is unique and remains the same throughout the trial.

### Sample size

The hypothesis of this study is that the use of chloroquine phosphate instead of lopinavir/ritonavir will increase the rate of throat-swab SARS-CoV-2 nucleic-acid negative conversion.

The main therapeutic index of this study is the clinical recovery time (after no more than 28 days), which is from the beginning of the study drug intervention treatment to the normalization of body temperature, respiratory symptoms, respiratory rate, and blood-oxygen saturation. In the later analysis, the log-rank method is used to compare the differences in clinical recovery time between the two groups of patients. The sample size of this study is calculated based on the log-rank method by using the log-rank test (Lakatos) (median survival time) module in the PASS 11.0 statistical software (see Additional file: Sample size report” for details).

Based on clinical experience [4, 18], the median clinical recovery time of the patients in the control group is expected to be 8 days, and the median clinical recovery time of patients in the experimental group can be shortened to 4 days (corresponding hazard ratio (HR) = 2.0) 112 patients (56 in each group) will be required to detect this difference with a significant level of *α* = 0.05 (both sides) with 85% confidence interval.

The trial is planned to be enrolled for 90 days, followed up for 28 days, and a final analysis is performed after 78 clinical recovery events occur. It is estimated that the drop-out rate of the experimental group and the control group is 5%.

### Statistical analysis

The results of this study for efficacy outcomes will be analyzed based on both intention-to-treat (ITT) and per-protocol (PP) approaches with a primary consideration for ITT results. A PP analysis will be performed secondarily. A safety analysis will be performed based on the safety group.

The ITT group will include participants who are randomized after satisfying the eligibility criteria and receive one study drug at least once and have post-dose evaluation data. The PP group will include participants who satisfy the following conditions among the ITT group: (1) those who completed all planned visits and (2) those who did not receive and use drugs or treatments that may affect the evaluation of efficacy during the study. The safety analysis group will include participants who received study drugs at least once and have post-dose safety evaluation data.

### Efficacy outcomes

Comparisons will be performed using two-sided tests with a statistical significance level of 5% unless stated otherwise.

#### Analysis of primary outcomes

For the primary outcome of this trial, the clinical recovery time of no more than 28 days after the completion of therapy and follow-up will be estimated as the proportion with a 95% confidence interval (CI) for each treatment group. The difference between the control arm and the experimental arm will subsequently be determined using the log-rank test. In order to control the influence of possible confounding factors such as gender and age on the clinical recovery time, the Cox proportional hazard model will be used to compare whether the clinical recovery time of the two groups is different, provide HRs and 95% CIs.

#### Analysis of secondary outcomes

The analysis of secondary outcomes will be described as explorative outcomes. The rate of treatment success after the completion of therapy and follow-up among the two groups will be compared using the chi-square test or Fisher’s exact test. The time of treatment success after no more than 28 days, the time to normal temperature (calculated from the onset of illness) during the completion of therapy and follow-up will be calculated in each group and compared using the log-rank test.

The rate of SAEs during the completion of therapy and follow-up will be compared among the two groups using the chi-square test or Fisher’s exact test.

### Safety assessment

All AEs according to the Common Terminology Criteria for Adverse Events (CTCAE) will be collected and documented, regardless of severity, seriousness, or relationship to the study drug. We will summarize all AEs, include AE frequency and percentage, and 95% CIs and compare the occurrence rate of AEs in relationship to the study drug and the severity of the two arms using the chi-square test or Fisher’s exact test.

### Stratified analysis

Primary and secondary outcomes will be analyzed separately in participants with throat-swab SARS-CoV-2 RT-PCR-positive and throat-swab SARS-CoV-2 RT-PCR-positive COVID-19 infection.

### Data collection and management

Our study will use a paper version of the case report form (CRF) and establish a clinical research database to record all the information in the CRF. We will use the software Epidata 3.1 for double data entry and proofreading of data, as well as manual verification and system verification.

During the study, medical personnel not participating in this study will monitor this trial. Monitors will visit the database to monitor all aspects of the study including adherence to the protocol and good clinical practice, protection of participants, and data accuracy of the study.

### Supervision of the trial

The Office of the Clinical Research Center and the Medical Ethics Committee of Sun Yat-sen University form the Data and Safety Monitoring Board. Based on data review during the trial conduct, the Board may provide recommendations such as protocol amendment, continuation, or stopping of the trial. The datasets analyzed during the current study are available from the corresponding author on reasonable request.

### Confidentiality

We will collect the participants’ personal information only when necessary to evaluate efficacy, safety, and tolerability of the study drugs. Such information will be collected and processed, taking precautions for compliance with laws on privacy protection and the guarantee of confidentiality. Paper files containing participants’ data (including personally identifiable information and copies of signed consent forms) will be securely stored in a locked office on sites in locked filing cabinets. Digital files containing participants’ data will be stored in password-protected files on university-maintained servers. Access to study files will be restricted to authorized personnel only.

The items in the present study protocol comply with the Standard Protocol Items: Recommendations for Interventional Trials (SPIRIT) Checklist (see the SPIRIT Checklist and figure in Additional file).

### Clinical trial registration

The trial was registered under the registration number ChiCTR2000029741 (http://www.chictr.org.cn/showproj.aspx?proj=49263) on 11 February 2020. On 10 February, 2020, this research was approved by the Medical Ethics Committee of the Fifth Affiliated Hospital of Sun Yat-sen University, ZDWY[2020] Lunzi No. (K15–1).

## Discussion

As of 10 April 2020, the total number of COVID-19 infection diagnoses in the world was more than 1.3 million, with more than 80,000 deaths (https://www.who.int/dg/speeches/detail/the-cooperation-council-of-the-turkic-speaking-states%2D%2D-10-april-2020). There is no known curative treatment for COVID-19, neither novel treatments nor vaccines. Cao’ study observed no benefit with lopinavir/ritonavir treatment in severe COVID-19 patients [19]. Lim’s study observed that the beta-coronavirus viral load significantly decreased with no or little coronavirus titers after administering lopinavir/ritonavir [2]. Gautret’s study [20] concluded that chloroquine is significantly associated with viral-load reduction/disappearance in COVID-19 patients. In the seventh edition of the Chinese version of the COVID-19 Diagnosis and Treatment Plan (http://www.nhc.gov.cn/yzygj/s7653p/202003/46c9294a7dfe4cef80dc7f5912eb1989/files/ce3e6945832a438eaae415350a8ce964.pdf), the recommended antiviral drugs are lopinavir/ritonavir, chloroquine phosphate and other drugs.

The Chinese have decreased the current epidemic situation in China by using the recommended drugs. The relief of the epidemic in most provinces of China has at least confirmed the effectiveness of the treatment to a certain extent, but further strong and effective, evidence-based data is needed.

The trial will be conducted in a clinical outpatient and inpatient setting by experienced clinicians, and participants will be recruited from the patient base of the other three hospitals participating in the trial. The purpose of this prospective, open-label, multicenter randomized controlled, comprehensive clinical study is to evaluate the efficacy and safety of chloroquine phosphate and lopinavir/ritonavir in patients with mild/general COVID-19 infection. The results of this study should provide meaningful information and evidence for clinical practice and should help to design a proven and reasonable RCT soon.

### Limitations

Randomized controlled studies still have some design limitations. First, the sample size we use is relatively small and the 28-day treatment period is short. Therefore, we will not be able to estimate possible relapses of pneumonia after long-term treatment. Second, the pathophysiology of novel coronavirus pneumonia has not been elucidated. As only clinician assessment is used (including lung computed tomography (CT) results and count of viral load), there is no objective indicator to judge the effect of treatment on COVID-19 infection. Finally, the follow-up period in this study was relatively short. In light of these limitations, we will develop a more reasonable treatment cycle and follow-up period to explore the efficacy of chloroquine in patients with COVID-19.

We also know that there will be many biases in the open trial, and we have taken a number of measures to control the possible bias in the trial, as follows:

(1) Strict exclusion criteria are formulated to effectively control other confounding factors that may affect the efficacy; (2) the trial uses random grouping to ensure that the two groups of patients are comparable; (3) before the patient signs the informed consent form, the researchers and the patients make full communication to ensure that the patients understand the entire trial content, and try to eliminate the impact of the patient’s psychological state on the trial effect; (4) the main and secondary indicators for evaluating the efficacy are objective indicators to avoid the influence of subjective factors; (5) before the start of the trial, the researchers will conduct unified system training to ensure the uniformity and correctness of data collection and index evaluation.

## Trial status

The trial was registered under the registration number ChiCTR2000029741(http://www.chictr.org.cn/showproj.aspx?proj=49263) on 11 February 2020. On 10 February 2020, this study was approved by the Medical Ethics Committee of the Fifth Affiliated Hospital of Sun Yat-sen University in Zhuhai, ZDWY[2020] Lunzi No. (K15–1). Unique Protocol ID: ZDWY.GRBK.011. Protocol version date: 7 February 2020. The first participant was randomized in February 2020, and recruitment is ongoing. It is estimated that the recruitment will be completed on 31 May 2020. The final results will be reported next year.

## Supplementary information

**Additional file 1.**

**Additional file 2.** Standard Protocol Items: Recommendations for Interventional Trials (SPIRIT) 2013 Checklist: recommended items to address in a clinical trial protocol and related documents.

## Data Availability

The datasets used or analyzed in the current study are available from the corresponding author on reasonable request.

## Abbreviations

ACE2: Angiotensin-converting enzyme 2
AE: Adverse event
COVID-19: Corona Virus Disease-2019
CI: Confidence interval
CRF: Clinical research flowchart
CTCAE: Common Terminology Criteria for Adverse Events
HCoVs: Human corona viruses
HIV: Human immunodeficiency virus
ITT: Intention-to-treat
MAPK: Mitogen-activated protein kinase
PP: Per-protocol
RT-PCR: Reverse transcription polymerase chain reaction
S protein: Spike protein
SAE: Severe adverse event
SARS-CoV: Severe Acute Respiratory Syndrome Corona Virus
