# Supplementary material for: Efficacy of chloroquine versus lopinavir/ritonavir in mild/general COVID-19 infection: a prospective, open-label, multicenter, randomized controlled clinical study
Source: Trials. 2020 Jul 8;21:622. doi: 10.1186/s13063-020-04478-w (PMC7341476; doi:10.1186/s13063-020-04478-w)
Supplement: Supplementary file 1 — Additional file 1. [file 13063_2020_4478_MOESM1_ESM.rtf]

	2020/4/20 8:17:31      1

Group Sequential Survival Power Analysis using Simulation
Scenario 1 Numeric Results for Group Sequential Testing Hazard 1 = Hazard 2.
Hypotheses: H0: Hazard1=Hazard2; H1: Hazard1<>Hazard2
Test Statistic: Logrank Test
Alpha-Spending Function: O'Brien-Fleming Analog
Beta-Spending Function: None
Futility Boundary Type: None
Number of Looks: 5
Simulations: 2000

Numeric Summary for Scenario 1

------------------ Power -------------------      -------------------------- Alpha ----------------------------
Value	95% LCL	95% UCL	Target	Actual	95% LCL	95% UCL	Beta
0.857	0.842	0.872	0.050	0.054	0.044	0.064	0.143

					Accrual	Accrual	Total
N1	N2	HR	T1	T2	Time	Pattern	Time
56	56	2.000	8.000	4.000	90.0	Equal	118.0

                                       ----------------- Noncompliance -----------------
Loss1	Loss2	Prop 1	T1	Prop 2	T2
0.050	0.050	0.000	1.000	0.000	1.000

Whole Study Averages

                                         ------------ Cum. Subject Time ------------
--- Study Length ---        --------- H0 ---------        --------- H1 ---------
H0	H1	G1	G2	G1	G2
116.9	85.0	404.5	403.9	329.8	209.4

------------------- Sample Size -------------------       --------------------- Events ---------------------
----------- H0 -----------        --------- H1 ---------       --------- H0 ---------        --------- H1 ---------
G1	G2	G1	G2	G1	G2	G1	G2
55.8	55.8	48.3	48.4	34.8	34.7	28.6	36.1

References
Klein, J.P.; Moeschberger, M.L.. 1997. Survival Analysis. Springer-Verlag. New York.
Piantadosi, S.. 2005. Clinical Trials, A Methodologic Perspective, 2nd Ed. John Wiley & Sons, Inc. New Jersey.
Devroye, Luc. 1986. Non-Uniform Random Variate Generation. Springer-Verlag. New York.
Matsumoto, M. and Nishimura,T. 1998. 'Mersenne twister: A 623-dimensionally equidistributed uniform
   pseudorandom number generator.' ACM Trans. On Modeling and Computer Simulations.
Jennison, C.; Turnbull, B.W. 2000. Group Sequential Methods with Applications to Clinical Trials. Chapman &
   Hall. Boca Raton, FL.

	2020/4/20 8:17:31      2

Group Sequential Survival Power Analysis using Simulation

Report Definitions
Power is the probability of rejecting a false null hypothesis at one of the looks. It is the total proportion
   of alternative hypothesis simulations that are outside the significance boundaries.
Power 95% LCL and UCL are the lower and upper confidence limits for the power estimate. The width of the
   interval is based on the number of simulations.
Target Alpha is the user-specified probability of rejecting a true null hypothesis. It is the total alpha
   spent.
Actual Alpha is the alpha level that was actually achieved by the experiment. It is the total proportion of
   the null hypothesis simulations that are outside the significance boundaries.
Alpha 95% LCL and UCL are the lower and upper confidence limits for the actual alpha estimate. The width of
   the interval is based on the number of simulations.
Beta is the probability of accepting a false null hypothesis. It is the total proportion of alternative
   hypothesis simulations that do not cross the significance boundaries.
N1 and N2 are the sample sizes of each group if the study reaches the final look.
HR is the hazard ratio h2/h1 = T1/T2 at which power is computed.
T1 is the median survival time of the control group. It is the median survival time that is simulated for both
   groups under H0, and for group 1 under H1.
T2 is the median survival time of the treatment group. It is the median survival time that is simulated for
   group 2 under H1.
Accrual Time is the time during which subjects are enlisted into the study. It is sometimes known as the
   enlistment period or recruitment period.
Accrual Pattern describes the distribution of accrual accross the Accrual Time.
Total Time is the total length of the study. It is the sum of the accrual time and the follow-up time.
Loss1 and Loss2 are the loss rates (lost to follow-up and right censored) for the control and treatment
   groups, respectively.
Noncompliance Proportion 1 and Noncompliance Proportion 2 are the proportion noncompliant in each time period
   for the control and treatment groups, respectively.
Noncompliance T1 and Noncompliance T2 are the noncompliance median survival times for the control and
   treatment groups, respectively.
For Whole Study Averages, H0 and H1 refer to the simulations under the null and altervative hypotheses,
   respectively.
G1 and G2 refer to the simulations for Groups 1 and 2, respectively.
Study Length is the average length of the study across all simulations. This length includes accrual time and
   follow-up time.
Cumulative Subject Time is the average total time of subject involvement. It is the average sum of survival
   times, including event survival times and censored survival times.
Sample size is the average number of subjects entering the study before study termination. If looks are made
   only after the full accrual period, these values will equal N1 and N2.
Events is the average number of events in the study before study termination.

Summary Statements
Group sequential trials with group sample sizes of 56 and 56 at the final look achieve 86%
power to detect a hazard ratio of 2.000 at the 0.054 significance level (alpha) using a
two-sided Logrank Test.


	2020/4/20 8:17:31      3

Group Sequential Survival Power Analysis using Simulation
Detailed Input

		Control	Trt			NComp	NComp	NComp	NComp
		Hazard	Hazard	Control	Trt	Prop	Hazard	Prop	Hazard
Time	Accrual	Rate	Rate	Loss	Loss	Control	Control	Trt	Trt
Period	Pattern	h1	h2	Ls1	Ls2	NCP1	NCh1	NCP2	NCh2
1	Equal (Uniform)	0.087	0.173	0.050	0.050	0.000	0.693	0.000	0.693
2	Equal (Uniform)	0.087	0.173	0.050	0.050	0.000	0.693	0.000	0.693
3	Equal (Uniform)	0.087	0.173	0.050	0.050	0.000	0.693	0.000	0.693
4	Equal (Uniform)	0.087	0.173	0.050	0.050	0.000	0.693	0.000	0.693
5	Equal (Uniform)	0.087	0.173	0.050	0.050	0.000	0.693	0.000	0.693
6	Equal (Uniform)	0.087	0.173	0.050	0.050	0.000	0.693	0.000	0.693
7	Equal (Uniform)	0.087	0.173	0.050	0.050	0.000	0.693	0.000	0.693
8	Equal (Uniform)	0.087	0.173	0.050	0.050	0.000	0.693	0.000	0.693
9	Equal (Uniform)	0.087	0.173	0.050	0.050	0.000	0.693	0.000	0.693
10	Equal (Uniform)	0.087	0.173	0.050	0.050	0.000	0.693	0.000	0.693
11	Equal (Uniform)	0.087	0.173	0.050	0.050	0.000	0.693	0.000	0.693
12	Equal (Uniform)	0.087	0.173	0.050	0.050	0.000	0.693	0.000	0.693
13	Equal (Uniform)	0.087	0.173	0.050	0.050	0.000	0.693	0.000	0.693
14	Equal (Uniform)	0.087	0.173	0.050	0.050	0.000	0.693	0.000	0.693
15	Equal (Uniform)	0.087	0.173	0.050	0.050	0.000	0.693	0.000	0.693
16	Equal (Uniform)	0.087	0.173	0.050	0.050	0.000	0.693	0.000	0.693
17	Equal (Uniform)	0.087	0.173	0.050	0.050	0.000	0.693	0.000	0.693
18	Equal (Uniform)	0.087	0.173	0.050	0.050	0.000	0.693	0.000	0.693
19	Equal (Uniform)	0.087	0.173	0.050	0.050	0.000	0.693	0.000	0.693
20	Equal (Uniform)	0.087	0.173	0.050	0.050	0.000	0.693	0.000	0.693
21	Equal (Uniform)	0.087	0.173	0.050	0.050	0.000	0.693	0.000	0.693
22	Equal (Uniform)	0.087	0.173	0.050	0.050	0.000	0.693	0.000	0.693
23	Equal (Uniform)	0.087	0.173	0.050	0.050	0.000	0.693	0.000	0.693
24	Equal (Uniform)	0.087	0.173	0.050	0.050	0.000	0.693	0.000	0.693
25	Equal (Uniform)	0.087	0.173	0.050	0.050	0.000	0.693	0.000	0.693
26	Equal (Uniform)	0.087	0.173	0.050	0.050	0.000	0.693	0.000	0.693
27	Equal (Uniform)	0.087	0.173	0.050	0.050	0.000	0.693	0.000	0.693
28	Equal (Uniform)	0.087	0.173	0.050	0.050	0.000	0.693	0.000	0.693
29	Equal (Uniform)	0.087	0.173	0.050	0.050	0.000	0.693	0.000	0.693
30	Equal (Uniform)	0.087	0.173	0.050	0.050	0.000	0.693	0.000	0.693
31	Equal (Uniform)	0.087	0.173	0.050	0.050	0.000	0.693	0.000	0.693
32	Equal (Uniform)	0.087	0.173	0.050	0.050	0.000	0.693	0.000	0.693
33	Equal (Uniform)	0.087	0.173	0.050	0.050	0.000	0.693	0.000	0.693
34	Equal (Uniform)	0.087	0.173	0.050	0.050	0.000	0.693	0.000	0.693
35	Equal (Uniform)	0.087	0.173	0.050	0.050	0.000	0.693	0.000	0.693
36	Equal (Uniform)	0.087	0.173	0.050	0.050	0.000	0.693	0.000	0.693
37	Equal (Uniform)	0.087	0.173	0.050	0.050	0.000	0.693	0.000	0.693
38	Equal (Uniform)	0.087	0.173	0.050	0.050	0.000	0.693	0.000	0.693
39	Equal (Uniform)	0.087	0.173	0.050	0.050	0.000	0.693	0.000	0.693
40	Equal (Uniform)	0.087	0.173	0.050	0.050	0.000	0.693	0.000	0.693
41	Equal (Uniform)	0.087	0.173	0.050	0.050	0.000	0.693	0.000	0.693
42	Equal (Uniform)	0.087	0.173	0.050	0.050	0.000	0.693	0.000	0.693
43	Equal (Uniform)	0.087	0.173	0.050	0.050	0.000	0.693	0.000	0.693
44	Equal (Uniform)	0.087	0.173	0.050	0.050	0.000	0.693	0.000	0.693
45	Equal (Uniform)	0.087	0.173	0.050	0.050	0.000	0.693	0.000	0.693
46	Equal (Uniform)	0.087	0.173	0.050	0.050	0.000	0.693	0.000	0.693
47	Equal (Uniform)	0.087	0.173	0.050	0.050	0.000	0.693	0.000	0.693
48	Equal (Uniform)	0.087	0.173	0.050	0.050	0.000	0.693	0.000	0.693
49	Equal (Uniform)	0.087	0.173	0.050	0.050	0.000	0.693	0.000	0.693
50	Equal (Uniform)	0.087	0.173	0.050	0.050	0.000	0.693	0.000	0.693


	2020/4/20 8:17:31      4

Group Sequential Survival Power Analysis using Simulation
Detailed Input

		Control	Trt			NComp	NComp	NComp	NComp
		Hazard	Hazard	Control	Trt	Prop	Hazard	Prop	Hazard
Time	Accrual	Rate	Rate	Loss	Loss	Control	Control	Trt	Trt
Period	Pattern	h1	h2	Ls1	Ls2	NCP1	NCh1	NCP2	NCh2
51	Equal (Uniform)	0.087	0.173	0.050	0.050	0.000	0.693	0.000	0.693
52	Equal (Uniform)	0.087	0.173	0.050	0.050	0.000	0.693	0.000	0.693
53	Equal (Uniform)	0.087	0.173	0.050	0.050	0.000	0.693	0.000	0.693
54	Equal (Uniform)	0.087	0.173	0.050	0.050	0.000	0.693	0.000	0.693
55	Equal (Uniform)	0.087	0.173	0.050	0.050	0.000	0.693	0.000	0.693
56	Equal (Uniform)	0.087	0.173	0.050	0.050	0.000	0.693	0.000	0.693
57	Equal (Uniform)	0.087	0.173	0.050	0.050	0.000	0.693	0.000	0.693
58	Equal (Uniform)	0.087	0.173	0.050	0.050	0.000	0.693	0.000	0.693
59	Equal (Uniform)	0.087	0.173	0.050	0.050	0.000	0.693	0.000	0.693
60	Equal (Uniform)	0.087	0.173	0.050	0.050	0.000	0.693	0.000	0.693
61	Equal (Uniform)	0.087	0.173	0.050	0.050	0.000	0.693	0.000	0.693
62	Equal (Uniform)	0.087	0.173	0.050	0.050	0.000	0.693	0.000	0.693
63	Equal (Uniform)	0.087	0.173	0.050	0.050	0.000	0.693	0.000	0.693
64	Equal (Uniform)	0.087	0.173	0.050	0.050	0.000	0.693	0.000	0.693
65	Equal (Uniform)	0.087	0.173	0.050	0.050	0.000	0.693	0.000	0.693
66	Equal (Uniform)	0.087	0.173	0.050	0.050	0.000	0.693	0.000	0.693
67	Equal (Uniform)	0.087	0.173	0.050	0.050	0.000	0.693	0.000	0.693
68	Equal (Uniform)	0.087	0.173	0.050	0.050	0.000	0.693	0.000	0.693
69	Equal (Uniform)	0.087	0.173	0.050	0.050	0.000	0.693	0.000	0.693
70	Equal (Uniform)	0.087	0.173	0.050	0.050	0.000	0.693	0.000	0.693
71	Equal (Uniform)	0.087	0.173	0.050	0.050	0.000	0.693	0.000	0.693
72	Equal (Uniform)	0.087	0.173	0.050	0.050	0.000	0.693	0.000	0.693
73	Equal (Uniform)	0.087	0.173	0.050	0.050	0.000	0.693	0.000	0.693
74	Equal (Uniform)	0.087	0.173	0.050	0.050	0.000	0.693	0.000	0.693
75	Equal (Uniform)	0.087	0.173	0.050	0.050	0.000	0.693	0.000	0.693
76	Equal (Uniform)	0.087	0.173	0.050	0.050	0.000	0.693	0.000	0.693
77	Equal (Uniform)	0.087	0.173	0.050	0.050	0.000	0.693	0.000	0.693
78	Equal (Uniform)	0.087	0.173	0.050	0.050	0.000	0.693	0.000	0.693
79	Equal (Uniform)	0.087	0.173	0.050	0.050	0.000	0.693	0.000	0.693
80	Equal (Uniform)	0.087	0.173	0.050	0.050	0.000	0.693	0.000	0.693
81	Equal (Uniform)	0.087	0.173	0.050	0.050	0.000	0.693	0.000	0.693
82	Equal (Uniform)	0.087	0.173	0.050	0.050	0.000	0.693	0.000	0.693
83	Equal (Uniform)	0.087	0.173	0.050	0.050	0.000	0.693	0.000	0.693
84	Equal (Uniform)	0.087	0.173	0.050	0.050	0.000	0.693	0.000	0.693
85	Equal (Uniform)	0.087	0.173	0.050	0.050	0.000	0.693	0.000	0.693
86	Equal (Uniform)	0.087	0.173	0.050	0.050	0.000	0.693	0.000	0.693
87	Equal (Uniform)	0.087	0.173	0.050	0.050	0.000	0.693	0.000	0.693
88	Equal (Uniform)	0.087	0.173	0.050	0.050	0.000	0.693	0.000	0.693
89	Equal (Uniform)	0.087	0.173	0.050	0.050	0.000	0.693	0.000	0.693
90	Equal (Uniform)	0.087	0.173	0.050	0.050	0.000	0.693	0.000	0.693
91	100% Accrual	0.087	0.173	0.050	0.050	0.000	0.693	0.000	0.693
92	100% Accrual	0.087	0.173	0.050	0.050	0.000	0.693	0.000	0.693
93	100% Accrual	0.087	0.173	0.050	0.050	0.000	0.693	0.000	0.693
94	100% Accrual	0.087	0.173	0.050	0.050	0.000	0.693	0.000	0.693
95	100% Accrual	0.087	0.173	0.050	0.050	0.000	0.693	0.000	0.693
96	100% Accrual	0.087	0.173	0.050	0.050	0.000	0.693	0.000	0.693
97	100% Accrual	0.087	0.173	0.050	0.050	0.000	0.693	0.000	0.693
98	100% Accrual	0.087	0.173	0.050	0.050	0.000	0.693	0.000	0.693
99	100% Accrual	0.087	0.173	0.050	0.050	0.000	0.693	0.000	0.693
100	100% Accrual	0.087	0.173	0.050	0.050	0.000	0.693	0.000	0.693


	2020/4/20 8:17:32      5

Group Sequential Survival Power Analysis using Simulation
Detailed Input

		Control	Trt			NComp	NComp	NComp	NComp
		Hazard	Hazard	Control	Trt	Prop	Hazard	Prop	Hazard
Time	Accrual	Rate	Rate	Loss	Loss	Control	Control	Trt	Trt
Period	Pattern	h1	h2	Ls1	Ls2	NCP1	NCh1	NCP2	NCh2
101	100% Accrual	0.087	0.173	0.050	0.050	0.000	0.693	0.000	0.693
102	100% Accrual	0.087	0.173	0.050	0.050	0.000	0.693	0.000	0.693
103	100% Accrual	0.087	0.173	0.050	0.050	0.000	0.693	0.000	0.693
104	100% Accrual	0.087	0.173	0.050	0.050	0.000	0.693	0.000	0.693
105	100% Accrual	0.087	0.173	0.050	0.050	0.000	0.693	0.000	0.693
106	100% Accrual	0.087	0.173	0.050	0.050	0.000	0.693	0.000	0.693
107	100% Accrual	0.087	0.173	0.050	0.050	0.000	0.693	0.000	0.693
108	100% Accrual	0.087	0.173	0.050	0.050	0.000	0.693	0.000	0.693
109	100% Accrual	0.087	0.173	0.050	0.050	0.000	0.693	0.000	0.693
110	100% Accrual	0.087	0.173	0.050	0.050	0.000	0.693	0.000	0.693
111	100% Accrual	0.087	0.173	0.050	0.050	0.000	0.693	0.000	0.693
112	100% Accrual	0.087	0.173	0.050	0.050	0.000	0.693	0.000	0.693
113	100% Accrual	0.087	0.173	0.050	0.050	0.000	0.693	0.000	0.693
114	100% Accrual	0.087	0.173	0.050	0.050	0.000	0.693	0.000	0.693
115	100% Accrual	0.087	0.173	0.050	0.050	0.000	0.693	0.000	0.693
116	100% Accrual	0.087	0.173	0.050	0.050	0.000	0.693	0.000	0.693
117	100% Accrual	0.087	0.173	0.050	0.050	0.000	0.693	0.000	0.693
118	100% Accrual	0.087	0.173	0.050	0.050	0.000	0.693	0.000	0.693


Look Details for Scenario 1

                               ---- Sample Size ----    ----------------------- Events ----------------------   -- Cum. Subject Time --
Look	Time	Group 1	Group 2	Group 1	% of S.S.	Group 2	% of S.S.	Group 1	Group 2
1	23.6	14.7	14.6	6.5	44.5	9.2	44.5	74.8	53.0
2	47.2	29.2	29.4	15.6	53.4	20.5	53.4	180.0	119.2
3	70.8	44.0	44.2	24.9	56.5	31.9	56.5	286.4	185.3
4	94.4	56.0	56.0	33.7	60.2	42.5	60.2	388.9	246.1
5	118.0	56.0	56.0	35.1	62.8	43.3	62.8	406.1	250.7

Look Details Definitions (Averages are across all simulations)
Look is the number of the look.
Time is the Time of the look from the beginning of the study (i.e., the beginning of the accrual period).
Sample Size Group 1 is average number of individuals in the control group that have entered the study by the
   corresponding look. If looks are made only after the full accrual period, these values will equal N1.
Sample Size Group 2 is average number of individuals in the treatment group that have entered the study by the
   corresponding look. If looks are made only after the full accrual period, these values will equal N2.
Events Group 1 is the average number of events in the control group up to the corresponding look.
% of S.S. is the average percent of the control group sample size for which an event has occurred up to the
   corresponding look. It is Group 1 Events / Group 1 Sample Size.
Events Group 2 is the average number of events in the treatment group up to the corresponding look.
% of S.S. is the average percent of the treatment group sample size for which an event has occurred up to the
   corresponding look. It is Group 2 Events / Group 2 Sample Size.
Cumulative Subject Time is the average total time of subject involvement up to the corresponding look for the
   control and treatment groups. It is the average sum of survival times, including event survival times and
   censored survival times.

	2020/4/20 8:17:32      6

Group Sequential Survival Power Analysis using Simulation


Boundaries for Scenario 1

               -- Significance Boundary --
	Z-Value	P-Value
Look	Scale	Scale
1	+/- 3.522	0.000
2	+/- 3.132	0.002
3	+/- 2.674	0.007
4	+/- 2.166	0.030
5	+/- 1.937	0.053

Boundaries Definitions
Look is the number of the look.
Significance Boundary Z-Value Scale is the value such that statistics outside this boundary at the
   corresponding look indicate termination of the study and rejection of the null hypothesis. They are
   sometimes called efficacy boundaries.
Significance Boundary P-Value Scale is the value such that P-Values outside this boundary at the corresponding
   look indicate termination of the study and rejection of the null hypothesis. This P-Value corresponds to
   the Z-Value Boundary and is sometimes called the nominal alpha.


	2020/4/20 8:17:32      7

Group Sequential Survival Power Analysis using Simulation
Boundary Plot


	2020/4/20 8:17:33      8

Group Sequential Survival Power Analysis using Simulation
Boundary Plot - P-Value


	2020/4/20 8:17:33      9

Group Sequential Survival Power Analysis using Simulation


Significance Boundaries with 95% Simulation Confidence Intervals for Scenario 1

                  --------- Z-Value Boundary ---------    --------- P-Value Boundary ---------
Look	Value	95% LCL	95% UCL	Value	95% LCL	95% UCL
1	+/- 3.522			0.000		
2	+/- 3.132			0.002		
3	+/- 2.674	-2.982	-2.486	0.007	0.003	0.013
4	+/- 2.166	-2.416	-2.087	0.030	0.016	0.037
5	+/- 1.937	-2.030	-1.869	0.053	0.042	0.062

Significance Boundary Confidence Limit Definitions
Look is the number of the look.
Z-Value Boundary Value is the value such that statistics outside this boundary at the corresponding look
   indicate termination of the study and rejection of the null hypothesis. They are sometimes called efficacy
   boundaries.
P-Value Boundary Value is the value such that P-Values outside this boundary at the corresponding look
   indicate termination of the study and rejection of the null hypothesis. This P-Value corresponds to the
   Z-Value Boundary and is sometimes called the nominal alpha.
95% LCL and UCL are the lower and upper confidence limits for the boundary at the given look. The width of the
   interval is based on the number of simulations.

	2020/4/20 8:17:33      10

Group Sequential Survival Power Analysis using Simulation


Alpha-Spending and Null Hypothesis Simulation Details for Scenario 1

                                                         --------- Target ---------   --------- Actual ----------  Proportion          Cum.
				Cum.			H1 Sims	H1 Sims
	--- Signif. 	Boundary---	Spending	Spending		Cum.	Outside	Outside
	Z-Value	P-Value	Function	Function	Alpha	Alpha	Signif.	Signif.
Look	Scale	Scale	Alpha	Alpha	Spent	Spent	Boundary	Boundary
1	+/- 3.522	0.000	0.000	0.000	0.001	0.001	0.012	0.012
2	+/- 3.132	0.002	0.001	0.001	0.004	0.005	0.129	0.140
3	+/- 2.674	0.007	0.007	0.008	0.006	0.011	0.314	0.454
4	+/- 2.166	0.030	0.017	0.024	0.019	0.030	0.340	0.794
5	+/- 1.937	0.053	0.026	0.050	0.025	0.054	0.063	0.857

Alpha-Spending Details Definitions
Look is the number of the look.
Significance Boundary Z-Value Scale is the value such that statistics outside this boundary at the
   corresponding look indicate termination of the study and rejection of the null hypothesis. They are
   sometimes called efficacy boundaries.
Significance Boundary P-Value Scale is the value such that P-Values outside this boundary at the corresponding
   look indicate termination of the study and rejection of the null hypothesis. This P-Value corresponds to
   the Significance Z-Value Boundary and is sometimes called the nominal alpha.
Spending Function Alpha is the intended portion of alpha allocated to the particular look based on the
   alpha-spending function.
Cumulative Spending Function Alpha is the intended accumulated alpha allocated to the particular look. It is
   the sum of the Spending Function Alpha up to the corresponding look.
Alpha Spent is the proportion of the null hypothesis simulations resulting in statistics outside the
   Significance Boundary at this look.
Cumulative Alpha Spent is the proportion of the null hypothesis simulations resulting in Significance Boundary
   termination up to and including this look. It is the sum of the Alpha Spent up to the corresponding look.
Proportion H1 Sims Outside Significance Boundary is the proportion of the alternative hypothesis simulations
   resulting in statistics outside the Significance Boundary at this look. It may be thought of as the
   incremental power.
Cumulative H1 Sims Outside Significance Boundary is the proportion of the alternative hypothesis simulations
   resulting in Significance Boundary termination up to and including this look. It is the sum of the
   Proportion H1 Sims Outside Significance Boundary up to the corresponding look.

	2020/4/20 8:17:33      11

Group Sequential Survival Power Analysis using Simulation
Numeric Results for Group Sequential Testing Hazard 1 = Hazard 2.
Hypotheses: H0: Hazard1=Hazard2; H1: Hazard1<>Hazard2
Test Statistic: Logrank Test
Alpha-Spending Function: O'Brien-Fleming Analog
Beta-Spending Function: None
Futility Boundary Type: None
Number of Looks: 5
Simulations: 2000
Pool Size: 10000

Numeric Summary of Scenarios

								Accrual	Accrual	Total
Scenario	Power	N1	N2	Alpha	HR	T1	T2	Time	Pattern	Time
1	0.857	56	56	0.054	2.000	8.000	4.000	90.0	Equal	118.0

Loss and Noncompliance Summary of Scenarios

                                                    ------------ Noncompliance ------------
Scenario	Loss1	Loss2	Rate 1	T1	Rate 2	T2
1	0.050	0.050	0.000	1.000	0.000	1.000

References
Klein, J.P.; Moeschberger, M.L.. 1997. Survival Analysis. Springer-Verlag. New York.
Piantadosi, S.. 2005. Clinical Trials, A Methodologic Perspective, 2nd Ed. John Wiley & Sons, Inc. New Jersey.
Devroye, Luc. 1986. Non-Uniform Random Variate Generation. Springer-Verlag. New York.
Matsumoto, M. and Nishimura,T. 1998. 'Mersenne twister: A 623-dimensionally equidistributed uniform
   pseudorandom number generator.' ACM Trans. On Modeling and Computer Simulations.
Jennison, C.; Turnbull, B.W. 2000. Group Sequential Methods with Applications to Clinical Trials. Chapman &
   Hall. Boca Raton, FL.

Report Definitions
Power is the probability of rejecting a false null hypothesis at one of the looks. It is the total proportion
   of alternative hypothesis simulations that are outside the significance boundaries.
Alpha is the alpha level that was actually achieved by the experiment. It is the total proportion of the null
   hypothesis simulations that are outside the significance boundaries.
N1 and N2 are the sample sizes of each group if the study reaches the final look.
HR is the hazard ratio h2/h1 = T1/T2 at which power is computed.
T1 is the median survival time of the control group. It is the median survival time that is simulated for both
   groups under H0, and for group 1 under H1.
T2 is the median survival time of the treatment group. It is the median survival time that is simulated for
   group 2 under H1.
Accrual Time is the time during which subjects are enlisted into the study. It is sometimes known as the
   enlistment period or recruitment period.
Accrual Pattern describes the distribution of accrual accross the Accrual Time.
Total Time is the total length of the study. It is the sum of the accrual time and the follow-up time.
Loss1 and Loss2 are the loss rates (lost to follow-up and right censored) for the control and treatment
   groups, respectively.
Noncompliance Proportion 1 and Noncompliance Proportion 2 are the proportion noncompliant in each time period
   for the control and treatment groups, respectively.
Noncompliance T1 and Noncompliance T2 are the noncompliance median survival times for the control and
   treatment groups, respectively.


Power and Alpha Summary

                     ------------------ Power -------------------      -------------------------- Alpha ----------------------------
Scenario	Value	95% LCL	95% UCL	Target	Actual	95% LCL	95% UCL	Beta
1	0.857	0.842	0.872	0.050	0.054	0.044	0.064	0.143

Report Definitions
Power is the probability of rejecting a false null hypothesis at one of the looks. It is the total proportion
   of alternative hypothesis simulations that are outside the significance boundaries.
Power 95% LCL and UCL are the lower and upper confidence limits for the power estimate. The width of the
   interval is based on the number of simulations.
Target Alpha is the user-specified probability of rejecting a true null hypothesis. It is the total alpha
   spent.
Alpha or Actual Alpha is the alpha level that was actually achieved by the experiment. It is the total
   proportion of the null hypothesis simulations that are outside the significance boundaries.
Alpha 95% LCL and UCL are the lower and upper confidence limits for the actual alpha estimate. The width of
   the interval is based on the number of simulations.
Beta is the probability of accepting a false null hypothesis. It is the total proportion of alternative
   hypothesis simulations that do not cross the significance boundaries.


Sample Size Summary
                                                                                                 ------- Average Sample Size -------
                                                                                                 --- Given H0 ---     --- Given H1 ---
Scenario	Power	Alpha	N1	N2	Grp1	Grp2	Grp1	Grp2
1	0.857	0.054	56	56	116.9	85.0	90.0	118.0

Report Definitions
Power is the probability of rejecting a false null hypothesis at one of the looks. It is the total proportion
   of alternative hypothesis simulations that are outside the significance boundaries.
Alpha is the alpha level that was actually achieved by the experiment. It is the total proportion of the null
   hypothesis simulations that are outside the significance boundaries.
N1 and N2 are the sample sizes of each group if the study reaches the final look.
Average Sample Size Given H0 Grp1 and Grp2 are the average or expected sample sizes of each group if H0 is
   true. These are based on the proportion of null hypothesis simulations that cross the significance or
   futility boundaries at each look.
Average Sample Size Given H1 Grp1 and Grp2 are the average or expected sample sizes of each group if H1 is
   true. These are based on the proportion of alternative hypothesis simulations that cross the significance
   or futility boundaries at each look.

Run Time: 51.41 seconds.


	2020/4/20 8:17:34      12

Group Sequential Survival Power Analysis using Simulation
Chart Section
